# Supplementary material for: Genetic Complexity in Spondyloarthritis: Contributions of HLA-B Alleles Beyond HLA-B*27 in Romanian Patients
Source: Int J Mol Sci. 2025 Aug 6;26(15):7617. doi: 10.3390/ijms26157617 (PMC12347857; doi:10.3390/ijms26157617)
Supplement: Supplementary file 1 [file ijms-26-07617-s001.zip › Supplementary Tables S1-S4.pdf]

Table S1. Distribution of HLA-B alleles in the entire group, and among male and female populations.

HLA-B	Gene Frequency	Total Number of Patients	Male	Female	*p*-value
**B*07**	16%	37	11	26	0,150
**B*08**	23%	47	18	29	0,628
**B*13**	12%	28	9	19	0,315
**B*14**	4%	10	3	7	0,528
**B*15**	8%	19	10	9	0,343
**B*18**	28%	59	31	28	0,072
**B*35**	35%	75	33	42	0,679
**B*37**	2%	4	1	3	0,642
**B*38**	12%	26	7	19	0,142
**B*39**	5%	11	4	7	0,766
**B*40**	9%	17	4	13	0,133
**B*41**	6%	12	9	3	0,032
**B*44**	18%	39	16	23	1
**B*47**	5%	12	4	8	0,766
**B*48**	1%	3	2	1	0,573
**B*49**	5%	11	8	3	0,057
**B*50**	4%	9	6	3	0,171
**B*51**	18%	39	14	25	0,484
**B*52**	6%	12	5	7	1
**B*53**	2%	4	1	3	0,642
**B*54**	1%	2	1	1	1
**B*55**	3%	7	1	6	0,245
**B*56**	2%	5	1	4	0,404
**B*57**	4%	9	6	3	0,117
**B*58**	3%	6	4	2	0,406

Table S2. Distribution of HLA-B alleles according to clinical features in the HLA-B*27-negative SpA cohort. Values represent the percentage of patients with a given clinical manifestation who carried the respective allele.

HLA-B	Low back pain (%)	Reduced lumbar mobility (%)	Arthritis (%)	Dactylitis (%)	Enthesitis (%)	IBD (%)	Uveitis (%)	Psoriasis (%)	Radiographical sacroiliitis (%)	Elevated CRP/ESR levels (%)
**B*07**	15,14	13,94	10,28	6,67	21,95	14,29	8,00	21,21	17,78	14,10
**B*08**	17,30	14,90	21,50	20,00	14,63	28,57	24,00	12,12	18,89	16,67
**B*13**	11,35	8,65	11,21	20,00	7,32	14,29	8,00	21,21	8,89	11,54
**B*14**	3,24	2,88	2,80	13,33	2,44	0,00	0,00	3,03	5,56	5,13
**B*15**	5,95	3,85	8,41	13,33	7,32	14,29	12,00	6,06	7,78	6,41
**B*18**	23,24	20,67	25,23	20,00	24,39	14,29	28,00	18,18	25,56	24,36
**B*35**	29,19	21,63	28,97	26,67	24,39	28,57	24,00	33,33	23,33	34,62
**B*37**	1,08	0,96	1,87	13,33	0,00	0,00	0,00	3,03	1,11	1,28
**B*38**	11,89	7,21	9,35	0,00	12,20	14,29	4,00	6,06	6,67	7,69
**B*39**	3,78	3,37	5,61	6,67	4,88	0,00	4,00	6,06	3,33	6,41
**B*40**	7,57	6,73	7,48	20,00	4,88	0,00	8,00	0,00	7,78	6,41
**B*41**	3,24	3,85	5,61	6,67	2,44	0,00	0,00	6,06	4,44	3,85
**B*44**	12,97	12,98	14,95	13,33	9,76	14,29	16,00	21,21	13,33	10,26
**B*47**	4,32	4,33	5,61	0,00	7,32	0,00	8,00	3,03	4,44	2,56
**B*48**	1,08	0,96	0,93	0,00	2,44	0,00	0,00	0,00	0,00	2,56
**B*49**	4,32	3,85	4,67	0,00	2,44	0,00	0,00	3,03	4,44	3,85
**B*50**	3,24	2,88	0,00	0,00	4,88	14,29	8,00	0,00	5,56	5,13
**B*51**	12,97	12,02	14,95	13,33	19,51	28,57	24,00	9,09	12,22	17,95
**B*52**	5,95	5,77	2,80	0,00	2,44	0,00	0,00	6,06	5,56	0,00
**B*53**	1,62	0,96	0,93	0,00	0,00	0,00	0,00	0,00	1,11	2,56
**B*54**	1,08	0,96	0,00	0,00	0,00	0,00	0,00	3,03	1,11	0,00
**B*55**	2,16	2,40	2,80	6,67	0,00	0,00	0,00	0,00	2,22	1,28
**B*56**	2,16	1,92	0,93	0,00	2,44	0,00	8,00	3,03	2,22	0,00
**B*57**	3,24	2,40	2,80	0,00	0,00	0,00	4,00	12,12	6,67	3,85
**B*58**	2,16	1,92	2,80	0,00	4,88	0,00	0,00	3,03	2,22	2,56

Table S3. Associations of HLA-B alleles with clinical manifestations in the HLA-B*27-negative SpA cohort (Fisher's Test)

HLA-B	*p*-value									
	**Low back pain**	**Reduced lumbar mobility**	**Arthritis**	**Dactylitis**	**Enthesitis**	**IBD**	**Uveitis**	**Psoriasis**	**Radiographical sacroiliitis**	**Elevated CRP/ESR levels**
**B*07**	0,561	0,129	0,154	0,702	0,140	1,000	0,547	0,280	0,262	1,000
**B*08**	0,726	1,000	0,251	0,736	0,661	0,612	0,412	0,470	0,738	0,861
**B*13**	0,665	0,221	0,840	0,205	0,588	0,550	1,000	0,062	0,674	0,827
**B*14**	0,489	0,433	0,745	0,105	1,000	1,000	0,605	1,000	0,318	0,489
**B*15**	0,295	0,033	0,630	0,296	1,000	0,412	0,404	1,000	0,805	1,000
**B*18**	0,747	0,822	0,371	1,000	0,839	1,000	0,458	0,658	0,436	0,630
**B*35**	0,766	0,130	0,890	1,000	0,578	1,000	0,816	0,539	0,197	0,179
**B*37**	0,584	0,188	1,000	0,017	1,000	1,000	1,000	0,417	1,000	1,000
**B*38**	0,115	1,000	0,537	0,375	0,572	0,522	0,485	0,754	0,277	0,505
**B*39**	0,737	1,000	0,363	0,483	0,683	1,000	1,000	0,634	0,754	0,311
**B*40**	0,410	0,210	0,616	0,063	1,000	1,000	0,669	0,141	0,600	1,000
**B*41**	0,191	0,275	0,555	0,513	0,698	1,000	0,612	0,651	1,000	1,000
**B*44**	0,254	0,670	0,860	1,000	0,472	1,000	0,773	0,294	0,716	0,190
**B*47**	0,754	0,716	0,555	1,000	0,407	1,000	0,319	1,000	1,000	0,519
**B*48**	1,000	0,500	1,000	1,000	0,400	1,000	1,000	1,000	0,553	0,211
**B*49**	1,000	1,000	0,762	1,000	1,000	1,000	0,607	1,000	1,000	1,000
**B*50**	0,727	1,000	0,012	1,000	0,634	0,219	0,207	0,608	0,282	0,457
**B*51**	0,254	0,670	1,000	1,000	0,346	0,278	0,231	0,436	0,466	0,349
**B*52**	0,117	0,134	0,370	1,000	0,698	1,000	0,612	0,651	0,551	0,021
**B*53**	1,000	1,000	0,648	1,000	1,000	1,000	1,000	1,000	1,000	0,584
**B*54**	1,000	1,000	0,516	1,000	1,000	1,000	1,000	0,236	1,000	1,000
**B*55**	0,427	0,635	1,000	0,340	0,600	1,000	1,000	0,601	1,000	0,678
**B*56**	1,000	1,000	0,651	1,000	0,575	1,000	0,073	0,491	1,000	0,326
**B*57**	0,727	0,691	0,742	1,000	0,362	1,000	0,599	0,017	0,067	0,727
**B*58**	1,000	1,000	0,690	1,000	0,236	1,000	1,000	0,556	1,000	1,000

Table S4. Logistic regression analysis of HLA-B allele distribution by sex

HLA-B	*p*-value	OR	95% Confidence Interval	
			Lower	Upper
**B*07**	0,152	2,078	0,764	5,662
**B*08**	0,164	2,031	0,751	5,425
**B*13**	0,260	1,870	0,629	5,578
**B*14**	0,250	2,565	0,517	12,663
**B*15**	0,800	0,775	0,262	2,809
**B*18**	0,431	0,726	0,285	1,707
**B*35**	0,948	1,046	0,425	2,494
**B*37**	0,156	7,020	0,477	101,353
**B*38**	0,083	2,838	0,873	9,047
**B*39**	0,545	1,577	0,362	6,836
**B*40**	0,126	2,991	0,735	12,214
**B*41**	0,044	0,200	0,041	0,961
**B*44**	0,699	1,214	0,456	3,224
**B*47**	0,448	1,723	0,420	7,094
**B*48**	0,566	0,493	0,038	6,021
**B*49**	0,062	0,221	0,045	1,078
**B*50**	0,189	0,341	0,065	1,716
**B*51**	0,455	1,446	0,545	3,885
**B*52**	0,892	0,914	0,230	3,590
**B*53**	0,352	3,119	0,283	34,661
**B*54**	0,924	0,855	0,043	17,197
**B*55**	0,217	4,176	0,432	40,253
**B*56**	0,217	5,005	0,389	64,010
**B*57**	0,277	0,406	0,080	2,059
**B*58**	0,150	0,233	0,032	1,697
